# Mirror symmetry and aging: The role of stimulus figurality and attention to colour

**DOI:** 10.3758/s13414-022-02565-5

**Published:** 2022-09-29

**Authors:** Jasna Martinovic, Jonas Huber, Antoniya Boyanova, Elena Gheorghiu, Josephine Reuther, Rafael B. Lemarchand

**Affiliations:** 1 Department of Psychology, School of Philosophy, Psychology and Language Sciences, University of Edinburgh, 7 George Square, Edinburgh, Scotland EH8 9JZ UK; 2 School of Psychology, University of Aberdeen, Aberdeen, UK; 3 University College London, London, UK; 4 Department of Psychology, Faculty of Natural Sciences, University of Stirling, Stirling, UK; 5 Department of Experimental Psychology, University of Göttingen, Göttingen, Germany

**Keywords:** Symmetry, Attention, Colour, Ageing, Perceptual organization

## Abstract

Symmetry perception studies have generally used two stimulus types: figural and dot patterns. Here, we designed a novel figural stimulus—a wedge pattern—made of centrally aligned pseudorandomly positioned wedges. To study the effect of pattern figurality and colour on symmetry perception, we compared symmetry detection in multicoloured wedge patterns with nonfigural dot patterns in younger and older adults. Symmetry signal was either segregated or nonsegregated by colour, and the symmetry detection task was performed under two conditions: with or without colour-based attention. In the first experiment, we compared performance for colour-symmetric patterns that varied in the number of wedges (24 vs. 36) and number of colours (2 vs. 3) and found that symmetry detection was facilitated by attention to colour when symmetry and noise signals were segregated by colour. In the second experiment, we compared performance for wedge and dot patterns on a sample of younger and older participants. Effects of attention to colour in segregated stimuli were magnified for wedge compared with dot patterns, with older and younger adults showing different effects of attention to colour on performance. Older adults significantly underperformed on uncued wedge patterns compared with dot patterns, but their performance improved greatly through colour cueing, reaching performance levels similar to young participants. Thus, while confirming the age-related decline in symmetry detection, we found that this deficit could be alleviated in figural multicoloured patterns by attending to the colour that carries the symmetry signal.

**Supplementary Information:**

The online version contains supplementary material available at 10.3758/s13414-022-02565-5.

## Introduction

Mirror symmetry is ubiquitous in the natural world. It occurs when two halves of a pattern mirror each other across a symmetry axis. Mirror symmetry detection is an effortless process when compared with detection of translational or rotational symmetry (Julesz, 1971). Studies have shown that a 50-ms exposure time is enough to discriminate mirror symmetry from noise, highlighting the speed and efficiency with which the human visual system can processes mirror symmetry (C. C. Chen & Tyler, 2010). However, the visual system is also sensitive to the colours rather than just positions of features, symmetric or otherwise. Symmetry sometimes correlates with colour and brightness modulations within objects such as fruit, flowers, human and animal faces, and bodies. While the role of patterns’ element position in mirror symmetry perception has been extensively investigated (for a review, see Bertamini et al., 2018), the extent to which colour and luminance polarity inconsistency of the symmetric elements affects symmetry detection is still debated.

A number of studies have investigated the role of colour in symmetry perception (Gheorghiu et al., 2016; Morales & Pashler, 1999; Wright et al., 2018; Wu & Chen, 2014, 2017). Initially, Morales and Pashler (1999) used two and four-colour nonisoluminant patterns in which the elements (squares) were arranged either symmetric or asymmetric (i.e., mismatched in colour) and found that symmetry detection mechanisms are not colour selective. An increase in the number of colours (from two to four) resulted in longer symmetry detection times and lower accuracy, which led the authors to propose that symmetry in multi-colour patterns could only be detected by a sequential attention-switching mechanism from one colour to the next. Connecting these ideas with feature integration model of attention (1980), Huang and Pashler (2002) further suggested that symmetry may be assessed one colour at a time using internal representations which specify the spatial distribution of a particular feature as either present or absent (e.g., red and nonred). For images that contain more than two colours, colour–symmetry would thus have to be serially evaluated.

On the other hand, Wu and Chen (2014) claimed that symmetry detection was colour selective based on the elevation of symmetry detection thresholds by a noise mask when noise and symmetric elements were of the same chromaticity. Gheorghiu et al. (2016) clarified whether symmetry channels were colour selective or colour sensitive by studying symmetry detection in random dot patterns in which colour and symmetry signals were either correlated or uncorrelated. Measuring symmetry detection under two perceptual conditions—“ with” and “without attention” to colour—Gheorghiu et al. showed that while symmetry detection mechanisms are sensitive to colour correlations across the symmetry axis and benefit from attention to colour, they are not colour selective (i.e., there are no colour-tuned symmetry channels). The effects reported in Wu and Chen’s (2014) study can be explained by the fact that participants knew the colour of the symmetric pattern, and thus they could selectively attend to the colour carrying the symmetry signal helping them to segregate symmetry from noise and thus facilitating symmetry detection.

In nature, symmetry is a salient attribute of figures (e.g., flowers, animal bodies, faces) rather than backgrounds, leading to higher conspicuity of symmetric animals. To impede predator discrimination prey may exhibit background matching or disruptive colouration, with the latter being particularly effective at decreasing detectability when placed away from the animal’s midline (i.e., symmetry axis; Wainwright et al., 2020). On this basis, generalizability of results from random-dot symmetries has been questioned by some authors (e.g., Wilson & Wilkinson, 2002). How similar is the role of colour in symmetry perception for dot patterns compared with figural patterns? Random dot patterns isolate sensitivity to positional information but lack any orientation information. One could argue that this is an important attribute of symmetric figures in everyday life. Using stimuli made of Gabor patches, Machilsen et al. (2009) found that orientation noise decreases the salience of symmetrical contour shapes embedded in backgrounds. Meanwhile, Sharman and Gheorghiu (2019) showed that orientation of elements does not affect symmetry detection in Gabor patterns if these are not embedded in a background, suggesting that symmetry detection mechanisms are solely reliant on positional information. However, neither of these studies examined the role of colour. Therefore, it remains unknown whether colour–symmetry correlations would become more conspicuous in figural stimuli containing both positional and orientation cues.

**Fig. 1:**
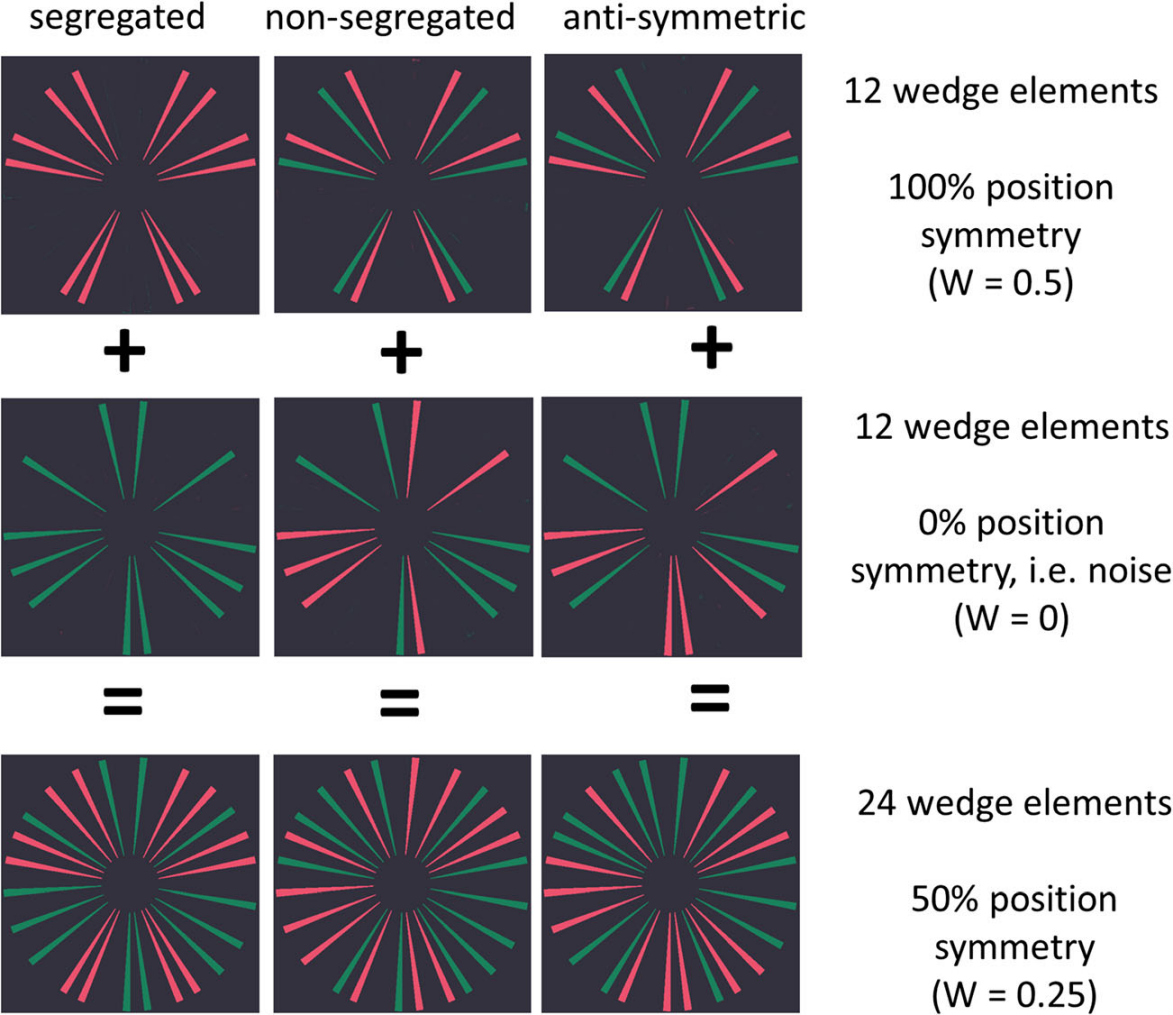
Properties of colour-symmetric wedge patterns. An example of how a 24-wedge pattern is built by combining twelve 100% positionally symmetric wedge elements and 12 noise elements. Each wedge pattern contains a symmetry signal (i.e., 100% position symmetry, as reflected by the weight of evidence W score of perceptual goodness equal to 0.5 meaning that there are 6 symmetric pairs out of a total of 12 elements) and noise (0% position symmetry). This results in a symmetric pattern with 50% position symmetry for the two-colour condition (W = 0.25) and 33% position symmetry (W = 0.17) for the three-colour condition. There are three colour arrangement conditions: segregated (left), nonsegregated (middle), and antisymmetric (right), all containing 50% position symmetry. In the segregated condition, the symmetry signal is of a single colour (either red or green) and noise of another colour (either green or red). In the nonsegregated condition, the symmetry signal is distributed evenly across the two colours (both red and green) as with the noise. In the antisymmetric condition, the symmetric elements are made of both colours but with symmetric pairs having opposite colours across the symmetry axis. Note that the number of wedges in each colour is equal across the symmetry axis. (Colour figure online)

**Fig. 2:**
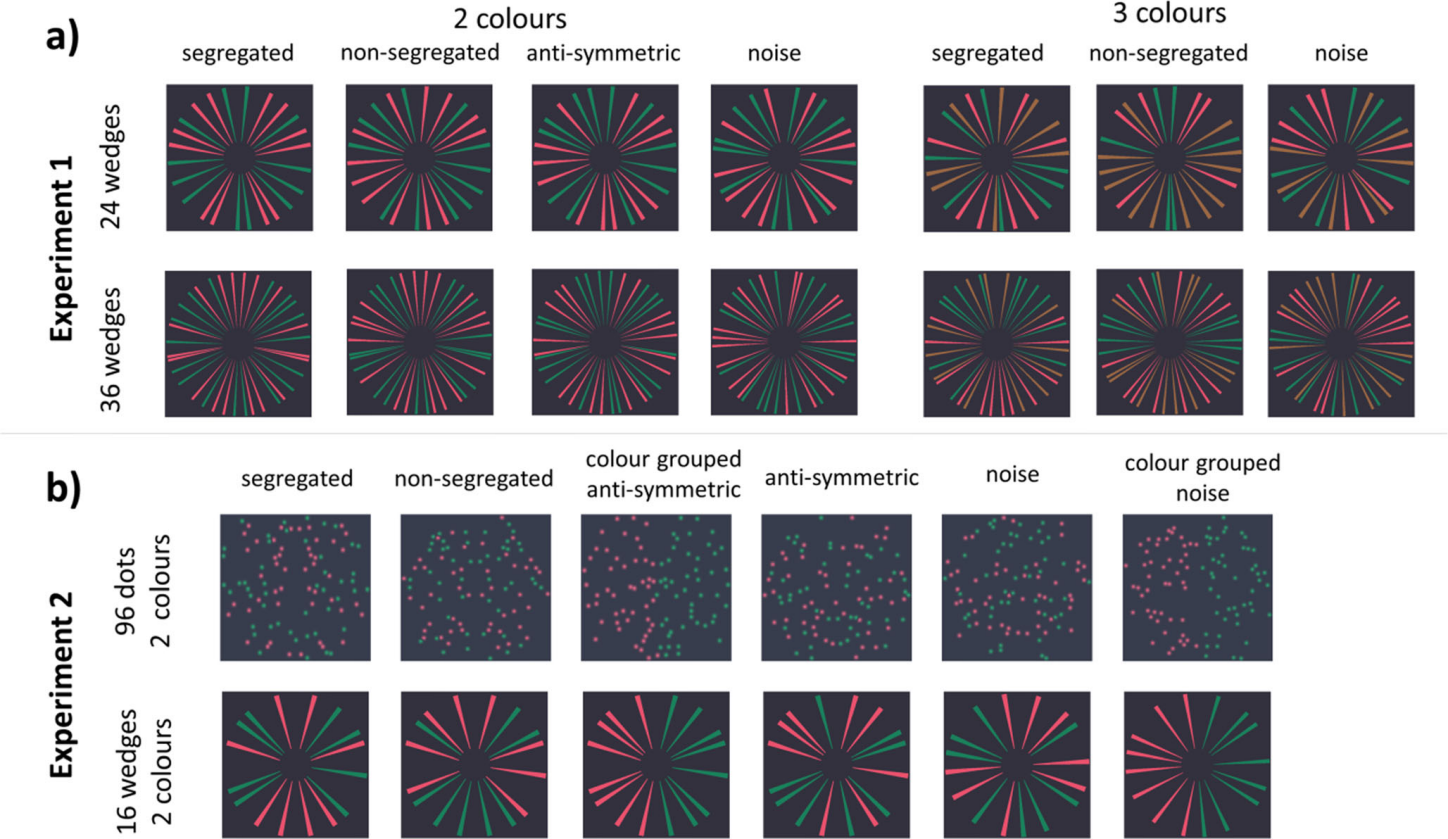
Example stimuli for the different colour–symmetry and noise combinations. **a** Experiment 1: Wedge stimuli consisted of 24 (top) or 36 (bottom) wedges and were of made of two (left) or three (right) colours. There were three colour–symmetry conditions: segregated, nonsegregated, and antisymmetric (see text and Fig. 1 for further details). **b** Experiment 2 contrasted performance for dot (top) and wedge (bottom) patterns using the segregated, nonsegregated, and antisymmetric conditions. The foil for these conditions was random distributed dots/wedges of all colours in equal proportions. In addition, we included a colour-grouped antisymmetric condition and a colour-grouped random pattern in which one side of the pattern was of one colour and the other side was of a different colour. The colour-grouped noise patterns served as foil for the colour-grouped antisymmetric condition. Note: the colour of the symmetry signal in the segregated conditions is red. (Colour figure online)

To address these outstanding issues, we used patterns made of Gaussian blobs similar to those used by Gheorghiu et al. (2016) and contrasted them with a novel wedge pattern made of centrally aligned but pseudorandomly positioned elements. While dot patterns allow for tight control over positional information in the absence of orientation, figural patterns contain both position and orientation information consistent with a figural object (see Fig. 1). As in Gheorghiu et al. (2016), we used a two-interval forced-choice (2IFC) task and measured accuracy for detecting symmetry in two-colour patterns containing 50% position symmetry and three-colour patterns containing 33% position symmetry under four stimulus conditions: (1) segregated patterns, in which symmetric and random (or noise) elements had different colours (e.g., all symmetric elements were red, and all random elements were green); (2) nonsegregated patterns, in which symmetric and random elements were of all colours in equal proportion (e.g., in two-colour patterns half of all symmetric and random elements were red, and the other half green); (3) antisymmetric patterns in which position-symmetric elements were mismatched in colour across the symmetry axis and random elements were assigned different colours in equal proportion across the symmetry axis; (4) colour-grouped antisymmetric patterns in which each half of the stimulus was of a different colour (Fig. 2).

Based on previous findings from Gheorghiu et al. (2016), we expected attention to colour to facilitate symmetry detection for segregated stimuli only (i.e., stimuli in which symmetry is carried by a single colour) but have no effect for other stimulus conditions in which symmetry is distributed across all colours. We expected attentional effects to be magnified for wedge patterns. While colour would attract spatial attention to dot and wedge position alike, wedge patterns would carry additional orientation-symmetry information to be processed. If higher perceptual load increases attentional effects (Lavie, 1995), this should not only produce larger benefits for colour-segregated patterns but also larger costs in performance for antisymmetric stimuli, through restricting symmetry analysis to a wholly uninformative part of the stimulus. In fact, when asked to detect symmetry in antisymmetric patterns, participants seem to be able to do so only under conditions in which information on spatial location is easily accessible (e.g., when there is low element density and high contrast homogeneity; Mancini et al., 2005). In nongrouped antisymmetric stimuli there may be fewer residual processing resources for detecting the positional symmetry carried by wedges depicted by the unattended colour. If this is the case, the complete lack of symmetry in elements depicted by the attended colour should lead to reduced or even at-chance performance.

To our knowledge, only one study by Herbert et al. (2002) examined symmetry detection in dot patterns in older adults and found a large reduction in sensitivity to symmetry among participants aged 60 and above (*d’* ~1–2). The information degradation hypothesis suggests that degraded perceptual signal inputs lead to perceptual processing errors, which in turn contribute to cognitive deficits in otherwise healthy older adults (for a review see Monge & Madden, 2016). Contour integration—a task that requires interelement grouping by orientation—also exhibits age-related costs in performance (Roudaia et al., 2008, 2013). On the other hand, global shape discrimination thresholds obtained with Glass patterns (which lack oriented lines) are similar in younger and older participants, with only a small reduction in sensitivity once noise dots are added (*d’* change of ~0.3; Norman & Higginbotham, 2020). Could feature-based attention alleviate at least some of the age-related deficits in symmetry perception? In the case of colour symmetry in segregated two-colour displays (Figs. 1 and 2), if attention was applied with optimal efficiency, directing attention to the colour of the symmetric elements would lead to maximal strength signal (i.e., fully filtering out all the distractor elements).

To evaluate our predictions, we conducted two experiments. In Experiment 1, we used our novel wedge patterns to examine how symmetry detection is affected by the number of wedges, number or colours, and colour distribution between symmetric and noise wedges. If wedge patterns containing both position and orientation symmetry engage the same mechanisms as dot patterns which contain only position symmetry, one would expect to obtain similar dependencies: (1) invariance to the number of elements as long as their density remains relatively low (e.g., Rainville & Kingdom, 2002, report a symmetry integration region of ~18 elements on average) and (2) improvements through feature-based attentional cueing when symmetry signal and noise elements are segregated by colour (Gheorghiu et al., 2016). In Experiment 2, we examined aging effects on symmetry detection by comparing performance for symmetric wedge patterns and symmetric dot patterns between younger and older participants. We expected to observe an age-related deficit in symmetry perception (Herbert et al., 2002), which should be alleviated through attentional deployment to colour in displays in which symmetry and noise elements were segregated by colour.

## Methods

### Participants

In Experiment 1, a total of 21 participants were tested, but three of them were excluded due to poor overall performance (below 55%). This left 18 participants in the sample (two males, age range: 19–29 years, *M* = 22, *SD* = 2). In Experiment 2, there were 26 participants (eight males): 14 younger adults (age range: 20–27 years; *M* = 22, *SD* = 2) and 12 older adults (age range: 60–69 years; *M* = 65, *SD* = 3). Psychophysics utilizes precise measurement techniques and focuses on effects that are generally large and stable across participants (e.g., Baker et al., 2018); thus, our sample sizes, which were no different from those in previous similar studies (e.g., Gheorghiu et al., 2016), were deemed adequate in this context (Lakens, 2022).

Participants were recruited amongst undergraduate and post-graduate students as well as members of the University of Aberdeen’s School of Psychology participant panel. They received either course credit or a monetary reimbursement to compensate them for their time and effort. Participants had normal or corrected-to-normal visual acuity and normal colour vision as assessed by the City University Colour Test (Fletcher, 1975). In Experiment 2, visual acuity was verified using a Snellen chart. Participants gave written informed consent. The research protocol was approved by the University of Aberdeen School of Psychology ethics committee and participants were treated in accordance with the Declaration of Helsinki (1964).

### Stimuli

Measurements of screen phosphors by SpectroCAL (Cambridge Research Systems, UK) were used in combination with CIE 1931 colour matching functions to ensure accurate colour reproduction. CRS toolbox for MATLAB was used to control stimulus presentation and collect responses, while CRS Colour toolbox (Westland et al., 2012) was used to generate the nonisoluminant colour stimuli.

Participants sat in a testing chamber at a viewing distance of 80 cm from the screen and responded via a button box (in Experiment 1, Cedrus RB-530, Cedrus Corporation, San Pedro, CA; in Experiment 2, CT-6 box, CRS, UK). In Experiment 1, stimuli were presented on Display++ screen (CRS, UK). In Experiment 2, stimuli were presented on a ViewSonic P227f monitor under the control of a Dell PC equipped with a dedicated visual-stimulus generator (ViSaGe; CRS, UK). The chromatic and luminance output of the monitor were calibrated prior to testing using a ColorCal2 (CRS, UK).

A mid-grey background (CIE 1931, *x* = 0.2848, *y* = 0.2932, Luminance 23 cd/m^2^) was used. We used the unique hues—red (0.3521, 0.2966, 49.887 cd/m^2^), green (0.2618, 0.3612, 49.887 cd/m^2^), and yellow (0.348, 0.364, 49.887 cd/m^2^) from the normative data set by Wuerger (2013). Stimuli consisted of wedge/dot patterns containing either two (red and green) or three (red, green, yellow) colours with respectively 50% and 33% wedges/dots arranged symmetrically in the symmetric condition, while the remaining wedges/dots were randomly positioned and drawn equally from the remaining colours (Fig. 1). In the two-colour patterns, 50% of the elements were green and 50% were red. In three-colour patterns, 33% of elements were depicted in each colour.

There were five stimulus conditions: (1) “segregated” condition, in which the symmetric dots or wedges were of one colour, and the random (or “noise”) dots/wedges were of the remaining colour(s); (2) “random-segregated” condition, which was the same as the segregated condition, except that the colour of the symmetric elements was randomly assigned to a different colour on each trial instead of being the same across the entire experiment; (3) “nonsegregated” condition, in which the symmetric elements were of all colours in equal proportion, as were the noise elements; (4) “antisymmetric” stimuli, in which position-symmetric dots were mismatched in colour across the symmetry axis; (5) “colour-grouped antisymmetric patterns” was an antisymmetric pattern in which all elements on one half of the pattern were of one colour, while the other half had a different colour. Such colour-grouped patterns could only be generated for two-colour stimuli. In the random dots and wedge patterns (0% symmetry signal), the noise dots/wedges were made of all colours in equal proportions. We also used a colour-grouped random pattern in which half of the random pattern was of one colour (either red or green) and the other half was of a different colour (either green or red). This was used for comparison with colour-grouped antisymmetric patterns.

The MATLAB scripts for generating wedge patterns can be found in our study’s online depository (https://osf.io/mf9ug/). The holographic model of regularity (van der Helm & Leeuwenberg, 1996) states that the weight of evidence for regularity in a pattern can be expressed as:

1 $$ W=\mathrm{E}/\mathrm{N} $$

where W = perceptual goodness, E = evidence for regularity, equaling the number of symmetric pairs and N = total amount of information. In our case, E would be the pairs of wedges that overlap across the vertical symmetry axis and N would be the total number of wedges. Perfect symmetry would have W = 0.5. For our two-colour symmetric patterns W = 0.25, for three-colour symmetric patterns W = 0.17 and for random patterns W = 0.

In Experiment 1, we contrasted performance for two- and three-coloured patterns consisting of 24 or 36 wedges. The wedges covered 50% of the area of a circle subtending 13.79° in diameter. To avoid visual discomfort resulting from too many wedges proximal to each other in the centre of the circle, an area 3.10° in diameter at the centre of the image was left blank (Figs. 1 and 2). We used three types of colour symmetry: segregated, nonsegregated, and antisymmetric.

While Experiment 1 focused on characterizing how performance driven by the novel wedge stimulus relates to number of wedges or colours in a sample of younger participants, Experiment 2 aimed to contrast it to symmetry perception from the more classical random dot patterns in younger and older adults. As older adults were expected to have poorer performance than younger adults, we needed to ensure that robustly above-chance performance could be obtained in both groups. Thus, we conducted pilots in which we manipulated the number and density of wedges until we were satisfied that our experiment would yield reliably above-chance performance in older adults. Another aim of the pilots was to ensure relatively similar performance for segregated dot and wedge patterns. This would make the findings from different colour–symmetry conditions more easily interpretable through providing a point of correspondence between the two stimulus types at the upper end of performance. The experiment used wedge patterns consisting of 16 elements, occupying 20% of the surface area of a circle subtending 14.74° in diameter (Fig. 2) and dot patterns similar to those used by Gheorghiu et al. (2016), consisting of 96 Gaussian blobs (0.41° diameter with a Gaussian size standard deviation factor of 5) spread over an area approx. 11° × 11° visual angle.

### Procedure

**Fig. 3:**
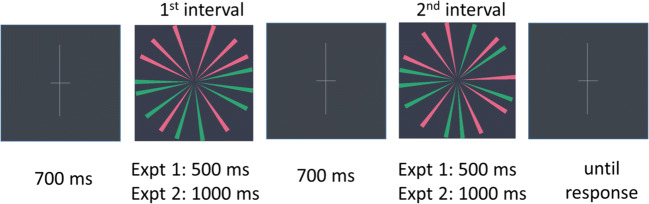
Schematic of the 2IFC procedure. In each trial, participants viewed two intervals—one containing a symmetric pattern and one showing a random/noise pattern (i.e., foil). The order of the patterns was randomized from trial to trial. In this example, the first interval contains the symmetric stimulus in which symmetry and noise are segregated by colour (symmetric wedges are all red), while the second interval contains a noise pattern (0% position symmetry). The fixation cross is elongated along the vertical axis to reinforce that this is the symmetry axis along which the circular patterns are to be judged. (Colour figure online)

We use a two-interval forced-choice (2IFC) procedure. At the start of each trial, a fixation cross would appear for 700 ms, and was followed by the sequential presentation of two images, one containing a symmetric pattern (i.e., either segregated, random segregated, nonsegregated, antisymmetric, or colour-grouped antisymmetric) and the other a random pattern (Fig. 3). The symmetric and random patterns were presented in random order and were separated by an inter-stimulus interval of 700 ms. The vertical line of the fixation cross was elongated (0.55° × 5.52°) to reinforce that mirror symmetry detection was to be performed across the vertical axis. Since the wedge-pattern was wheel-shaped, and wedge generation was evaluated with regard to vertical mirror symmetry, participants could potentially pick up on genuine, yet unintended symmetry if they were to evaluate the stimulus in relation to a different axis. Participant’s task was to indicate whether the symmetric pattern was in the first or second interval by using a key press. The left button corresponded to the first interval and the right button to the second. Participants were allowed to take as long as required to respond. In Experiment 1, stimulus images were presented for 500 ms (as in Gheorghiu et al., 2016) while in Experiment 2, presentation time was 1,000 ms. The longer stimulus duration in Experiment 2 was chosen following a pilot experiment and was intended to ensure that older adults could perform the symmetry detection task above chance level.

This experiment was repeated under two perceptual conditions: first, without attention to colour symmetry, and second, with attention to colour (i.e., participants were a priori told the colour of the symmetric pattern). Similar to Gheorghiu et al. (2016), in these attention-to-colour conditions the stimuli were not physically altered in any way, but participants were verbally informed of the symmetry colour to attend (i.e., the colour carrying the symmetry signal in the segregated condition). Half of subjects were cued to green, and the other half to red.

At the start of each block, participants first performed 16 practice trials, to get familiarized with the stimuli/task. Participants were asked to repeat the practice if they failed to exceed 60% accuracy. In Experiment 1, stimuli were blocked by number of wedges and the number of colours, with 120 trials per block (40 per colour–symmetry condition; segregated, nonsegregated, and antisymmetric). Block order was randomly assigned for each participant. In experiment 2, stimuli were blocked by stimulus type (wedges or dots), with 250 trials per block (50 per colour–symmetry condition; segregated, random segregated, nonsegregated, nongrouped antisymmetric, grouped antisymmetric). Block order was counterbalanced amongst participants, as was the attended colour. Breaks were offered between blocks. Experiment 1 took two hours to complete. Experiment 2 took two and a half hours to complete. Due to the lengthiness of Experiment 2, participants were offered the possibility to complete the study in two separate sessions.

### Statistical analysis

All analyses were performed in R (R Core Team, 2016), using packages *gtools* (Warnes et al., 2015), *reshape2* (Wickham, 2007), *dplyr* (Wickham et al., 2017), *ggplot2* (Wickham, 2009), *lme4* (Bates et al., 2015), *effectsize* (Ben-Shachar et al., 2020), and *emmeans* (Lenth et al., 2019).

The use of analysis of variance (ANOVA) is questionable when data outcomes are categorical (e.g., correct or incorrect response). Accuracy rates computed from single-trial data may appear suitable for an ANOVA but suffer from the problem that confidence intervals (CIs) may extend beyond interpretable values of 0 and 100% (e.g., a mean of 90% with a CI between 65% and 115%). While ordinary logit models have many advantages over ANOVAs on percentage data, mixed logit models have the further advantage of being able to account for random subject effects (Jaeger, 2008). We used generalized linear mixed-effect models (GLMMs) on the binomial single-trial accuracy data (correct/incorrect), as implemented in the R statistic package *lme4*. Effect sizes are reported as standardized odds ratios (*OR*s). An *OR* of ~1 would imply no difference in the likelihood of the two outcomes (here, correct or incorrect) between conditions, while *OR*s of 1.68, 3.47, and 6.71 can be taken as equivalent to Cohen’s *d* = 0.2 (small), 0.5 (medium), and 0.8 (large), respectively (H. Chen et al., 2010).

When fitting GLMMs, we applied the maximal random effect structure that was possible while maintaining goodness of fit. We then evaluated the contributions of fixed effects and their interactions by removing the highest order effects, one by one, and performing chi-squared tests to assess whether their removal affected the amount of variance explained. We report all the estimates of the final model, in which none of the remaining effects can be removed without reducing the variance explained. Post hoc tests on any interactions in this final model were performed using omnibus paired t tests, corrected for multiple comparisons (*p* < .05) with the “mvt” method from *emmeans* package, which relies on the multivariate *t* distribution with the same covariance structure as the estimates to determine the adjustment.

## Results

### Experiment 1: Symmetry detection in wedge patterns

We divided the dataset into two partly overlapping sets prior to submitting it to generalized linear mixed-effect model analysis. The first set allows us to examine the interplay between the number of wedges (24 or 36), number of colours (two or three), type of colour symmetry (segregated or nonsegregated), and attention (uncued or cued). The second set only includes two-coloured patterns, allowing us to examine the significance of the type of colour symmetry in more detail, by including antisymmetric in addition to segregated and non-segregated patterns.

**Fig. 4:**
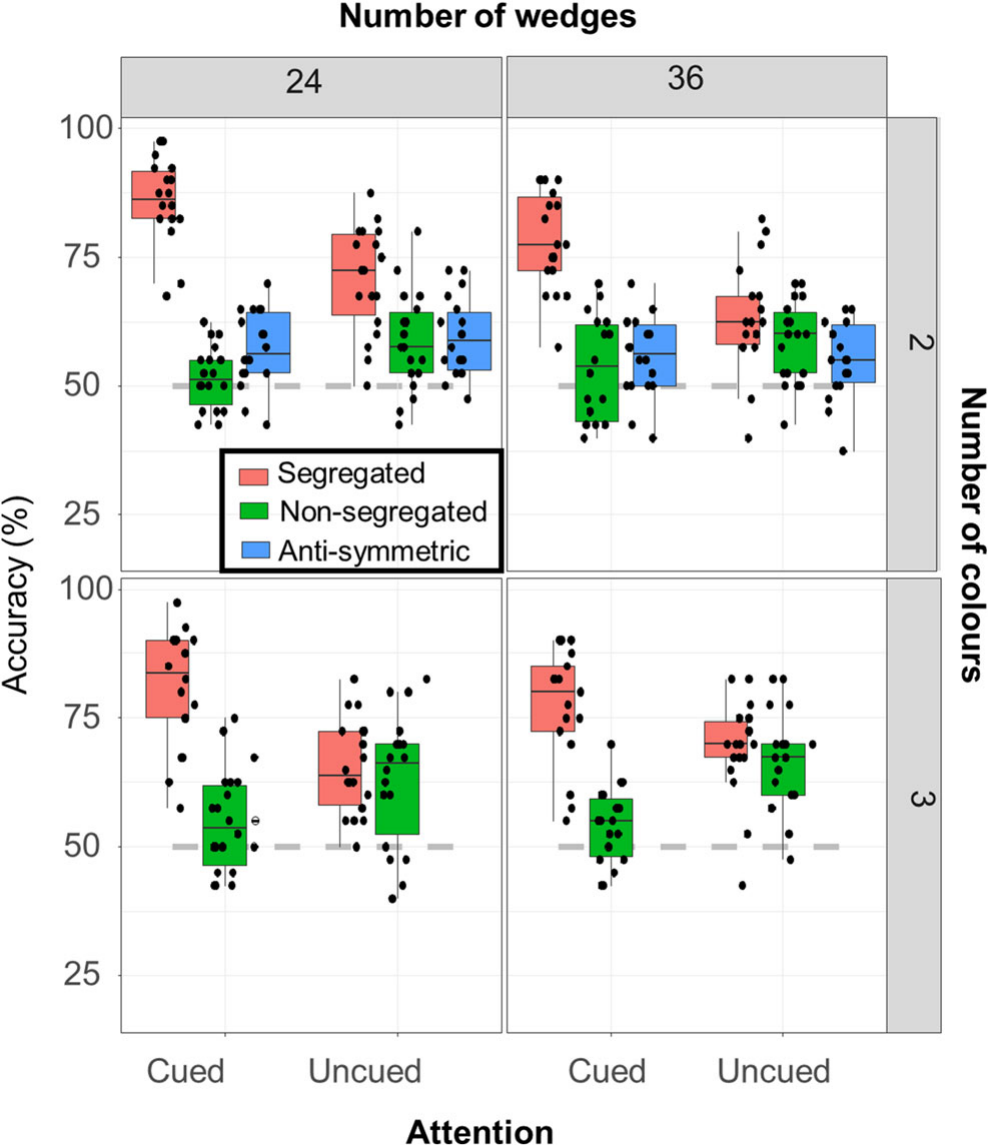
Results for Experiment 1: Box plot showing accuracy in the symmetry detection task. Dots indicate individual data points. The dashed grey line indicates the chance level (50% accuracy). (Colour figure online)

Figure 4 depicts accuracy as a function of stimulus type and attention condition (cued/uncued) for 24 and 36 number of wedges, and 2 and 3 number of colours conditions. Individual participant data points are overlaid onto the box plot. The full details of the best fitting model are presented in the Supplementary Materials.

For our first analysis, the best fitting model included the three-way interaction between number of colours, number of wedges and colour–symmetry type, χ^2^(1) = 5.481, *p* = .019, as well as two two-way interactions of attention, the first one with colour symmetry, χ^2^(1) = 22.198, *p* < .001, and the second one with number of colours, χ^2^(1) = 4.0161, *p* = .045. The four-way interaction, all the other three-way interactions and the two-way interaction between attention and number of wedges did not contribute to the fit and were thus removed (all *p*s > .095). For full details of the statistical tests, see the Supplementary Materials.

**Fig. 5:**
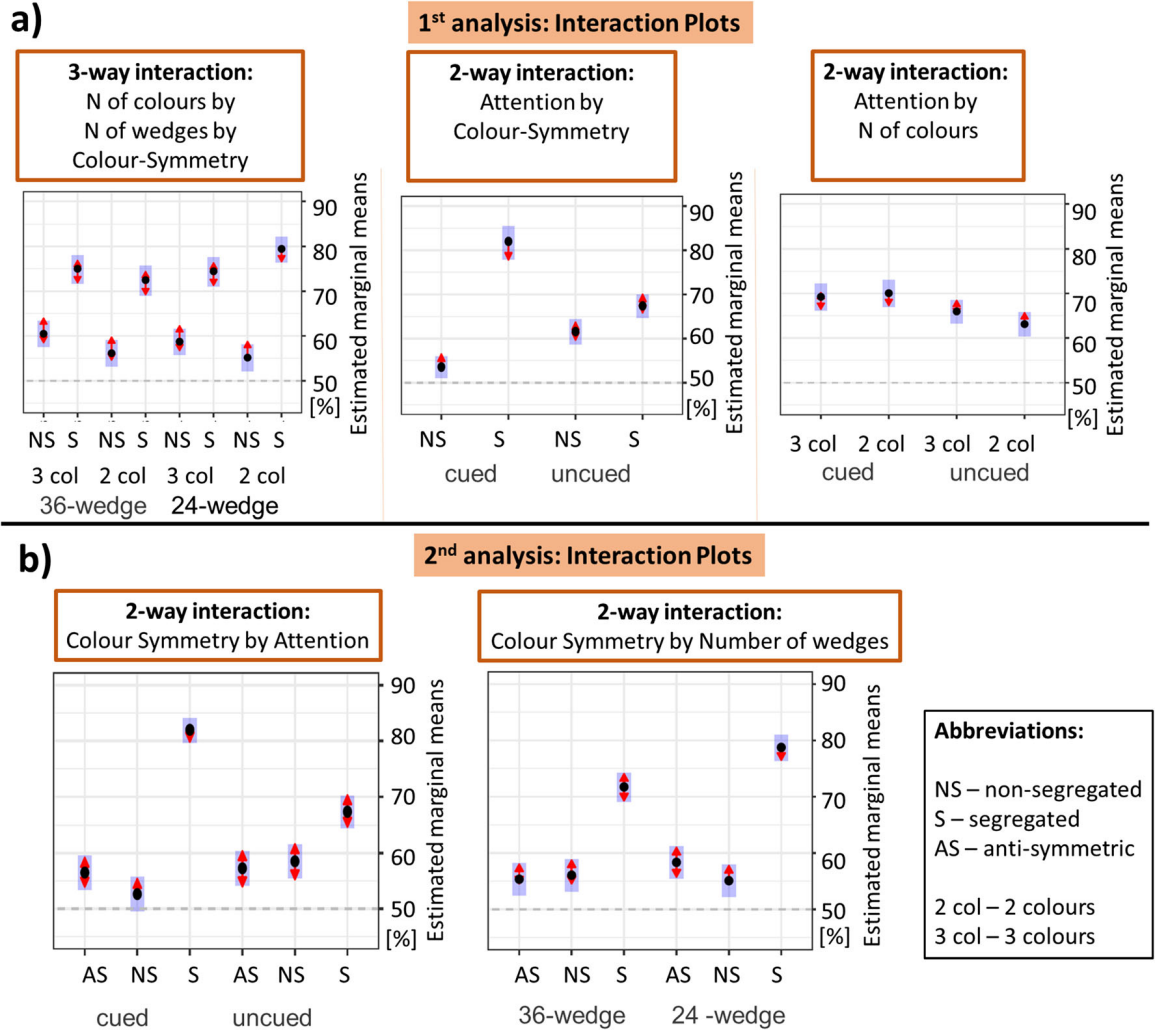
Plots of all the interactions from Experiment 1. **a** Interactions from the first analysis, which included patterns that differed in the number of colours. Estimated marginal means derived from the best fitting GLMM are presented on the *y*-axis, while the levels of factors involved in the interactions are presented on the *x*-axis. The other factors are collapsed. **b** Interaction plots for the second analysis involving all three symmetry conditions (nonsegregated, segregated and antisymmetric) for 24 or 36 wedges, with and without attention. Estimated response rates derived from the best fitting GLMM are shown on the *y*-axis, with only the factors involved in the interaction shown on the *x*-axis. Dashed lines indicate chance level, shaded blue areas indicate 95% confidence intervals of the estimate, and red arrows demarcate statistically significant differences (note that conditions for which the red errors overlap are not statistically different from each other). (Colour figure online)

In Fig. 5a, the left plot visualizes the three-way interaction between number of colours, number of wedges, and type of colour–symmetry patterns, the middle plot shows the two-way interaction between attention and type of colour–symmetry patterns and the right plot visualizes the interaction between attention and number of colours. In the segregated condition, performance for the two-colour 24-wedge patterns was significantly higher compared with both three-colour 24-wedge (*z* = 3.181, *p* = .029) and two-colour 36-wedge patterns (*z* = 4.373, *p* < .001). However, for non-segregated patterns performance did not depend on number of colours or wedges (*z* < 2.876, *p* > .072). Thus, two-colour 24-wedge patterns were not associated with better performance more generally, but only in the segregated condition. For the two-way interactions, significantly higher performance was obtained with-attention only in the segregated condition (*z* = 4.188, *p* < .001). Finally, number of colours was another factor that interacted with attention—while for two-colour patterns, attention did produce a significant increase in performance (*z* = 3.333, *p* = .004), this did not occur for three-colour patterns (*z* = −1.598, *p* = .360).

For our second analysis, we only examined two-colour patterns, allowing us to also include responses to antisymmetric patterns. Interactions between the number of wedges and colour–symmetry type, χ^2^(2) = 13.219, *p* = .001, and colour–symmetry type and attention, χ^2^(2) = 94.871, *p* < .001, contributed significantly to the model. All the other interactions were not significant (all *p*s > .511; for more detail, see the Supplementary Materials).

We evaluated the two interactions using omnibus paired *t* tests corrected for multiple comparisons, with interaction plots depicted in Fig. 5b (attention and colour–symmetry type on the left; number of wedges and colour–symmetry type on the right). In terms of the effect of attention to different types of colour symmetry, two things stand out: (1) while attention to colour creates a large benefit for segregated patterns (*z* = 8.109, *p* < .001), it does not have a pronounced effect on non-segregated (*z* = 2.781, *p* = .0593) or antisymmetric (*z* = 0.366, *p* = .99) patterns; (2) segregation produces a benefit even without cueing, with superior performance compared with uncued nonsegregated (*z* = 7.080, *p* < .001) and antisymmetric (*z* = 5.289, *p* < .001) patterns. Number of wedges interacted with different colour–symmetry types, so that performance was better for segregated 24-wedge patterns compared with 36-wedge patterns (*z* = 4.331, *p* < .001) but no significant differences were observed for nonsegregated (*z* = 0.527, *p* = .995) and antisymmetric patterns (*z* = 1.622, *p* = .583).

### Interim discussion

The results are highly consistent across the two analyses: attention improves performance for segregated patterns alone. Thus, we replicate the observations on colour symmetry reported by Gheorghiu et al. (2016) and extend them to figural patterns. The attentional improvement for segregated patterns occurs in addition to the existing but small advantage in relation to nonsegregated patterns. A similar benefit of attention when signal and noise elements are segregated by colour has also been found in other tasks such as global motion coherence (Li & Kingdom, 2001; Martinovic et al., 2009). The attentional benefit for segregated symmetric patterns is more pronounced in two-colour 24-wedge stimuli, indicating that a small number of colours and elements (low density) improves performance. A decrease in performance with number of colours in the stimuli was also reported by Gheorghiu et al. (2016). This is to be expected considering that the amount of symmetry signal decreases from 50% for two colours to 33% for three colours. It is important to note that overall performance in the experiment was relatively low (*M =* 62.9%, *SD* = 13.1%). Another factor potentially contributing to such low performance could have been the high density of wedge elements within the stimuli. Wedges covering 50% of the circle area left on average a 5° gap on the circumference between elements for 36-wedge patterns and a 7.5° gap between 24-wedge patterns. Higher density is associated with poorer symmetry detection as it leads to adoption of a smaller symmetry integration region and hence increases susceptibility to positional noise (Rainville & Kingdom, 2002).

This experiment established that symmetry detection in wedge patterns exhibits similar dependencies on stimulus properties (number of elements, colours, attention, and colour symmetry) as dot patterns. In the following experiment, we compare symmetry detection in wedge and dot pattern in younger and older adults. To increase overall performance, wedge patterns are reduced to 16 elements covering 20% of the circle (see Methods for more detail).

### Experiment 2: Symmetry detection for dot and wedge patterns in younger and older adults

**Fig. 6:**
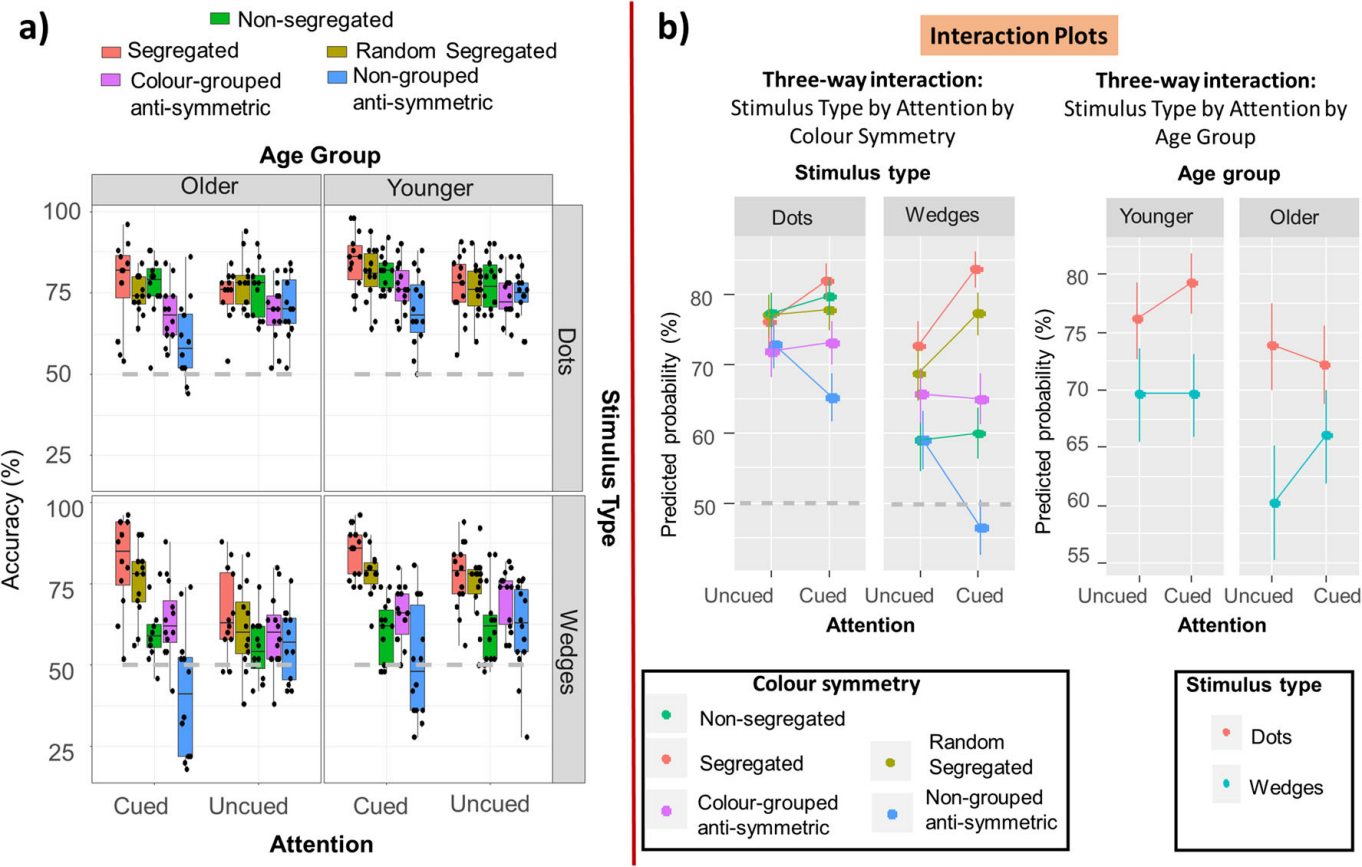
Results for Experiment 2. **a** Average accuracy in the symmetry detection task. Dots indicate individual participant data. The dashed grey line emphasizes 50% accuracy equivalent to chance performance. **b** Three-way interaction plots from the best fitting model, depicting data collapsed so as to visualize only those factors involved in the interactions. The left graph depicts the interaction between stimulus type, colour–symmetry and attention, collapsing across age. The right graph depicts the interaction between stimulus type, attention, and age, collapsing across colour–symmetry combinations. Model predictions are back transformed to reflect accuracy measures. Error bars depict 95% confidence intervals. (Colour figure online)

Figure 6a depicts average accuracy for older and younger participants for wedge and dot patterns in the symmetry detection task, with individual participant data (black dots) overlaid onto the graph. The full details of the best fitting model are presented in the Supplementary Materials.

The best fitting model included the following three-way interactions: Colour Symmetry × Stimulus Type (wedge, dots) × Attention, χ^2^(4) = 16.295, *p* = .003, and Stimulus Type × Attention × Age Group, χ^2^(1) = 20.303, *p* < .001. Figure 6b depicts the post hoc analyses, which focused on decomposing these two interactions.

First, we deconstructed the interaction between stimulus type, colour–symmetry type, and attention (see left panel in Fig. 6b). This interaction reveals that symmetry detection is not only more difficult in wedge stimuli but also associated with a somewhat different pattern of attentional effects for different colour–symmetry conditions. In fact, similar performance for wedges and dots in the segregated condition (both uncued and cued) despite a much poorer performance for the majority of other wedge-pattern conditions implies that colour–symmetry correlations may be more efficiently processed in wedge compared with dot patterns. More pronounced costs of cueing for nongrouped antisymmetric wedge patterns are in line with this account.

Overall, the post hoc analysis revealed some broad similarities in how cueing affected dots and wedges: segregated patterns were facilitated by attentional cueing (dots: *z* = 3.419, *p* = .012; wedges *z* = 6.265, *p* < .001), while nongrouped antisymmetric patterns were inhibited/hindered by feature-based attention (dots: *z* = 3.748, *p* = .003, wedges: *z* = 5.697, *p* < .001). In addition, cueing also increased performance for random segregated patterns, but only for wedge patterns (*z* = 4.522, *p* < .001). Effects of cueing were absent for all other colour–symmetry types (*z*s < 1.36, *p*s > .0947). Wedge and dot stimuli led to similar performance in the uncued segregated condition (*z* = 1.71, *p* = .769), which was unsurprising as our pilots aimed to match performance for this condition. There were no significant differences between uncued colour-grouped antisymmetric dot and wedge patterns (*z* = 2.886, *p* = .07). However, in all other uncued conditions, performance was poorer for wedges (random segregated: *z* = 4.288, *p* < .001; nonsegregated: *z* = 8.640, *p* < .001; nongrouped antisymmetric: *z* = 6.376, *p* < .001). When comparing cued wedge and dot patterns, performance remained poorer for nonsegregated (*z* = 9.450, *p* < .001) and nongrouped antisymmetric (*z* = 8.238, *p* < .001) wedges, became comparable for random segregated (*z* = 0.317, *p* = .990) and remained comparable for segregated (*z* = 1.119, *p* = .990) patterns.

Secondly, we deconstructed the interaction between stimulus type, attention and age group. Older and younger participants did not differ in uncued dots (*z* = 0.917, *p* = .940) or cued wedges (*z* = 1.298, *p* = .7541) conditions, but older adults performed poorer with uncued wedges (*z* = 2.884, *p* = .0325) and cued dots (*z* = 3.256, *p* = .0100). This shows that for older adults colour-based attentional cueing in dot patterns does not improve symmetry detection in a similar way as for younger observers, but it can improve their performance in wedge patterns, compared with their uncued performance.

## Discussion

This study characterizes colour–symmetry perception in younger and older adults using two types of stimuli: classical dot patterns and novel figural wedge patterns. Wedge patterns are constructed from centrally aligned but pseudorandomly positioned wedges, thus allowing for randomization of wedges locations/orientations. As in the previous work of Gheorghiu et al. (2016), we examine the relation between the position symmetry signal and colour symmetry, as well as the role of attention to colour. We replicate the findings that attention to colour increases accuracy when position and colour symmetry signals are correlated and decreases accuracy when colour is anti-correlated. Segregation of signal and noise by colour in wedge stimuli also leads to somewhat higher levels of performance even without cueing, in line with previous findings on automatic grouping-by-colour for weak motion signals (Martinovic et al., 2009). Finally, we also replicate a previous report of age-related costs to symmetry perception (Herbert et al., 2002). In addition, we find that older and younger adults show different effects of feature-based attention on performance. Older adults perform poorer when colour and symmetry are correlated but uncued in wedge patterns, but their performance with this stimulus improves greatly through colour cueing, reaching similar levels as in young participants. Meanwhile, younger adults benefit from attentional cueing of colour–symmetry correlations in dot patterns. However, a similar benefit from attentional cueing of symmetric dots’ colour fails to materialize in older adults. While confirming the age-related decline in symmetry perception, we thus also show that these costs can be overcome in figural colour–symmetric patterns by attending to the colour that carries the symmetry signal. The ability to group and attend signals based on their hue may thus be a key route to improving signal-to-noise ratio and overcoming age-related costs caused by noisier processing (Monge & Madden, 2016).

**Fig. 7:**
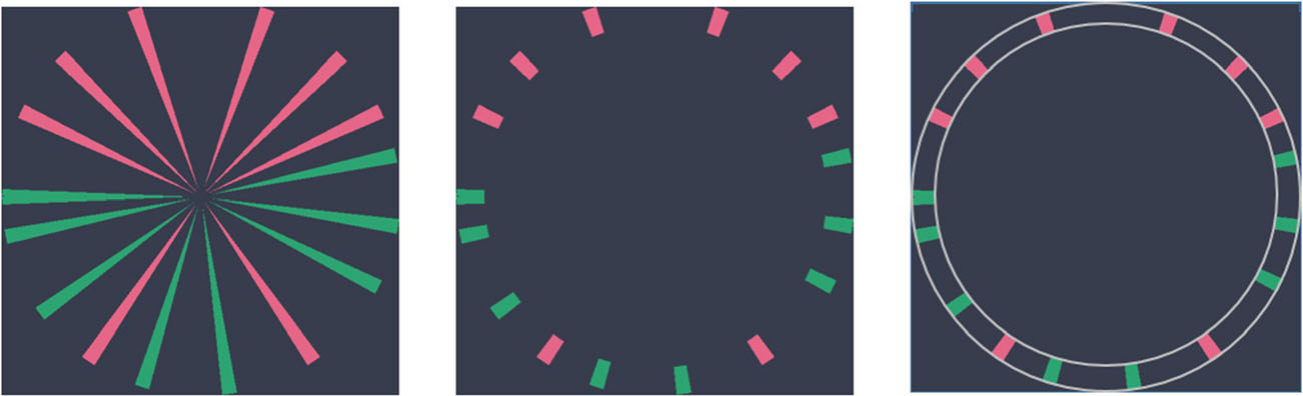
Wedge pattern (left), bar pattern with the segments arranged orthogonally to the imaginary circular path (centre), and ring-bar pattern (right) stimuli. Bar patterns can be created either from wedge patterns by removing figurality (global orientation information consistent with a figure/shape) through removal of the inner section of the circle (i.e., shortening the segments) or by symmetrically segmenting radial-frequency patterns such as those used by Wilson and Wilkinson (2002). Ring-bar patterns contain local orientation information embedded within a circular outline, making them more figural. Both bar and ring-bar patterns can be made of segments that are either collinear, orthogonal, or random to the imaginary circular contour path. They could be useful in future research investigating the interaction between position, orientation, length, and colour in symmetry perception. (Colour figure online)

While we replicate previously observed effects of attentional cueing to colour–symmetry correlations (Gheorghiu et al., 2016), we also find that such attentional effects are magnified for wedge patterns. Such benefits and costs of attention to colour of wedge patterns are in line with object-based accounts of attentional selection (Desimone & Duncan, 1995). However, there is another, more parsimonious explanation. In addition to positional information, wedge patterns also contain orientation information. This would introduce two sources of signal and noise—position and orientation (and/or segment length)—while dot patterns only contain position signals. Future work could also investigate how the length of the oriented wedge segments affect symmetry detection by making use of a coloured ringed stimulus to reduce orientation information and eliminate figurality (see Fig. 7). Such ring patterns could also be created by segmenting radial-frequency symmetric patterns (i.e., sinusoidally modulated circular patterns introduced by Wilson & Wilkinson, 2002). Those segments could be arranged either collinearly or orthogonally to the path of the imaginary symmetric contour. Computing spatial correspondences for short radial frequency segments (collinear/orthogonal) or ring-bar patterns should not be more difficult for the visual system than for dot patterns with the same positional information. As shape discrimination in older adults is similar to that of younger adults in the absence of noise (Norman & Higginbotham, 2020), we expect the same would be the case for symmetry discrimination when positional symmetry signal is 100%. By gradually adding noise to the signal elements, one could evaluate if symmetry perception in older adults is more susceptible to noise, similarly to global shape processing. If this is the case, then it would imply that increased susceptibility to noise in mid-level spatial integration mechanisms is an important driver of age-related costs in perceptual organization.

Bilaterally symmetric random dot patterns display an invariance to the number of elements as long as element density remains relatively low (e.g., Wenderoth, 1996). Rainville and Kingdom (2002) demonstrate that the spatial integration region for detecting symmetry is scale invariant, with 13–27 elements needed to perform the task successfully by different observers. The spatial extent of the wedge patterns has an additional constraint—they have to occupy a predefined area within a circle. The spatial extent of dot patterns is more arbitrary and can be more freely chosen by the experimenter. Rainville and Kingdom (2002) concluded that positional jitter can be tolerated until it exceeds the average spacing between elements. With 16, 24, and 36 wedge elements occupying 1° at circumference, this would mean that tolerance to noise would operate in average windows of 22.5°, 15°, and 10°. However, any increases in wedge size lead to a reduction of these windows. Of course, the same constraint would apply to the newly proposed bar and ring-bar patterns (see Fig. 7).

In this light, our first experiment set out to evaluate the influence of both the number of wedges and the number of colours on performance. While we do not find a main effect of wedge number, overall performance for 24 and 36 wedge patterns is reliably above chance but remains relatively low. There are also interactions of wedge number with attention and colour symmetry. Attention to correlated colour–symmetry patterns, in which signal and noise are segregated by colour, leads to biggest improvements in performance for two-coloured 24-wedge patterns. This could mean that attention is less able to overcome the costs introduced by increased jitter in the 36-wedge patterns or lower overall symmetry signal strength in three-colour patterns. Future studies should investigate the ability of attention-to-colour to improve grouping—with bilateral symmetry as a special case of this more general integrative process. This could be achieved by parametrically manipulating the amount of positional jitter in several steps, from 0% (all noise elements outside the tolerance region) to 100% (each noise element is within a signal element’s tolerance region).

Older adults may be particularly vulnerable to noise due to the degradation of sensory information brought about by healthy ageing (Monge & Madden, 2016). It has been argued that older adults also have specific difficulties when various competing regions need to be assigned figure or ground status during perceptual organization (Anderson et al., 2016; Lass et al., 2017). This is assumed to stem from deficits in inhibitory processing that are particularly pronounced when competition is high and in scenes that are more ambiguous or difficult to resolve. Our data is consistent with both the information degradation account and the reduced inhibition account, showing that in the absence of attentional cueing, younger and older adults perform similarly on nonsegregated dot patterns (78% and 76%, respectively), but younger adults perform better than older adults on the more challenging non-segregated wedge patterns (62% vs. 55%). The slightly worse performance in older than younger adults found in our study is consistent with reports from a single previous study on age-related changes in symmetry perception (Herbert et al., 2002). Further to that, we find differential effects of attention to colour in older and young adults. Older adults show a benefit from attention for wedge stimuli but not dot patterns. As mentioned earlier, figural wedges contain both position and orientation information, lowering uncued performance and hence creating plenty of room for potential attentional improvements. Our findings from Experiment 2 (Fig. 6) are consistent with this explanation—while younger and older adults perform similarly for uncued dot patterns, younger adults benefit from attention to colour in dot stimuli, while older adults fail to improve with attentional cueing. For wedge patterns, older adults perform poorer than younger adults but overcome this deficit through colour cueing.

Why would the benefits of attentional cueing dissociate between the two stimulus types in younger and older adults? Performance for wedge patterns is poorer in older adults, which in turn makes the colour cue more beneficial in guiding performance, similarly to Madden’s (1992) findings for spatial cueing. As colour–symmetry correlations modulate the efficiency of attentional cues differentially for dot and wedge stimuli, with magnified attentional effects for wedges, the superposition of these effects with ageing effects might lead to different outcomes when performance itself differs. Cueing can clearly lead to considerable improvements when starting from a relatively low wedge-symmetry detection level of ~60% in older adults. Here, benefits due to cueing can far surpass the more limited room for costs in nongrouped antisymmetric patterns. This would not be the case for younger adults, who already perform well above the chance level, and thus have less scope for attentional improvements due to colour–symmetry correlations. As performance is the outcome rather than a predictor, generalized linear mixed-effect models such as those fit in this study cannot capture these types of effects.

The lack of improvement in performance due to attentional cueing to features (colour) for older adults in dot patterns is more difficult to explain. It may be due to different feature-based attention strategies between younger and older adults, although Madden (1992) found the two age groups exhibit similar behaviour in terms of their use of focal and distributed *spatial* attention in a visual search task with spatial cues. In our colour-cued conditions, participants need to balance focusing their attention to the positions of elements depicted in the attended colour with distributing attention to positions of all the elements, as the cue is clearly not equally informative or useful in all trials. In spatial cueing, the cue can be valid if it points to the target location (e.g., left-pointing arrow, if the target is left), invalid if it points to a non-target location (e.g., right-pointing arrow), and neutral if it is uninformative about target location (e.g., double-sided arrow; see Posner et al., 1980). It could be argued that in our cued blocks, the colour cue is valid for the segregated condition and 50% of trials from random-segregated condition (i.e., 30% of total trials), as in those cases it correctly directs attention to the symmetry signal. In colour-grouped antisymmetric conditions (20% trials), the colour cue would be neutral as it would be clear to the participants that in order to detect symmetry they need to distribute their attention to elements on both sides of the vertical axis (i.e., across both colours). On nonsegregated trials, nongrouped antisymmetric trials and the remaining half of random-segregated trials (50% of the total) the colour cue would actually be invalid, directing participants’ attention away from some if not all of the symmetry signal. For example, when attending to one colour in the non-grouped antisymmetric condition, the antisymmetric pattern should appear equally symmetric as the fully random/noise pattern, and this is confirmed by at-chance performance for such antisymmetric wedge stimuli. Future studies should determine whether older and younger adults differ in their evaluation of featural cue validity (here, colour) and consequent strategic shifts between focal, feature-based, cue-driven attention and attention that is distributed equally across all the elements of a display. Older adults’ performance on perceptual and attentional tasks is known to be slower and more effortful (for an overview, see Monge & Madden, 2016). It is likely that such distinct performance parameters may also drive different attentional strategies, which would be yet another source of differences between younger and older adults, in addition to a general degradation of perceptual information or a specific reduction of inhibitory mechanisms proposed by existing models of healthy ageing (Betts et al., 2005; Monge & Madden, 2016; for attentional strategies and ageing see Vallesi et al., 2021).

Orientation information is highly relevant to shape perception in both younger and older participants (Roudaia et al., 2014), the latter of which exhibit poorer performance when asked to discriminate different noncircular patterns (*d’* reduction of ~0.7). Pilz et al. (2020) found a substantial age-related decline for oblique but not for cardinal orientations. This impairment in oblique orientation perception might have contributed to poorer symmetry-detection performance for wedge patterns in the older group in the absence of colour-based attention. Attention to colour could decrease the noisiness in symmetry detection in a similar way in which categorical representations (e.g., a cardinal orientation) provide more noise-resistant templates (see Lu & Dosher, 1998). This would lead to improved perceptual performance in older adults. Thus, the main practically relevant outcome of our study is that providing a priori knowledge about the features of objects (e.g., colour) may be a good heuristic for improving age-aware design: Through assisting older adults in selecting the dimension of interest when processing information in complex visual environments, their performance can be improved up to the level exhibited by younger observers.

## Supplementary information

ESM 1 (DOCX 24 kb)

## Data Availability

The study was not preregistered. Data, R analysis scripts, and MATLAB scripts for generating wedge patterns are available on Open Science Framework (https://osf.io/mf9ug/). For the purpose of open access, J.M. has applied a Creative Commons Attribution (CC BY) licence to any Author Accepted Manuscript version arising from this submission.

## References

[CR1] Anderson JA, Healey MK, Hasher L, Peterson MA (2016). Age-related deficits in inhibition in figure-ground assignment. Journal of Vision.

[CR2] Baker DH, Lygo FA, Meese TS, Georgeson MA (2018). Binocular summation revisited: Beyond √2. Psychological Bulletin.

[CR3] Bates D, Maechler M, Bolker B, Walker S (2015). Fitting linear mixed-effects models using lme4. Journal of Statistical Software.

[CR4] Ben-Shachar M, Lüdecke D, Makowski D (2020). effectsize: Estimation of effect size indices and standardized parameters. Journal of Open Source Software.

[CR5] Bertamini, M., Silvanto, J., Norcia, A. M., Makin, A. D. J., & Wagemans, J. (2018). The neural basis of visual symmetry and its role in mid- and high-level visual processing. *Annals of the New York Academy of Sciences*. 10.1111/nyas.13667 10.1111/nyas.13667 29604083

[CR6] Betts LR, Taylor CP, Sekuler AB, Bennett PJ (2005). Aging reduces center-surround antagonism in visual motion processing. Neuron.

[CR7] Chen CC, Tyler CW (2010). Symmetry: Modeling the effects of masking noise, axial cueing and salience. PLOS ONE.

[CR8] Chen H, Cohen P, Chen S (2010). How big is a big odds ratio? Interpreting the magnitudes of odds ratios in epidemiological studies. Communications in Statistics—Simulation and Computation.

[CR9] Desimone R, Duncan J (1995). Neural mechanisms of selective visual attention. Annual Review of Neuroscience.

[CR10] Fletcher R (1975). *The City University colour vision test*.

[CR11] Gheorghiu E, Kingdom FAA, Remkes A, Li HCO, Rainville S (2016). The role of color and attention-to-color in mirror-symmetry perception. Scientific Reports.

[CR12] Herbert AM, Overbury O, Singh J, Faubert J (2002). Aging and bilateral symmetry detection (Proceedings paper). Journals of Gerontology Series B—Psychological Sciences and Social Sciences.

[CR13] Huang L, Pashler H (2002). Symmetry detection and visual attention: A “binary-map” hypothesis. Vision Research.

[CR14] Jaeger TF (2008). Categorical data analysis: Away from ANOVAs (transformation or not) and towards logit mixed models. Journal of Memory and Language.

[CR15] Julesz B (1971). *Foundations of cyclopean perception*.

[CR16] Lakens D (2022). Sample size justification. Collabra: Psychology.

[CR17] Lass JW, Bennett PJ, Peterson MA, Sekuler AB (2017). Effects of aging on figure-ground perception: Convexity context effects and competition resolution. Journal of Vision.

[CR18] Lavie N (1995). Perceptual load as a necessary condition for selective attention. Journal of Experimental Psychology: Human Perception and Performance.

[CR19] Lenth, R. V., Singmann, H., Love, J., Buerkner, P., & Herve, M. (2019). emmeans: Estimated Marginal means, aka least squares means (Version 1.4.3.01) [Computer software]*.* https://CRAN.R-project.org/package=emmeans

[CR20] Li H-CO, Kingdom FAA (2001). Segregation by colour/luminance does not necessarily facilitate motion discrimination in noise. Perception & Psychophysics.

[CR21] Lu Z-L, Dosher BA (1998). External noise distinguishes attention mechanisms. Vision Research.

[CR22] Machilsen B, Pauwels M, Wagemans J (2009). The role of vertical mirror symmetry in visual shape detection. Journal of Vision.

[CR23] Madden DJ (1992). Selective attention and visual search: Revision of an allocation model and application to age differences. Journal of Experimental Psychology: Human Perception and Performance.

[CR24] Mancini S, Sally SL, Gurnsey R (2005). Detection of symmetry and anti-symmetry. Vision Research.

[CR25] Martinovic J, Meyer G, Muller MM, Wuerger SM (2009). S-cone signals invisible to the motion system can improve motion extraction via grouping by color. Visual Neuroscience.

[CR26] Monge ZA, Madden DJ (2016). Linking cognitive and visual perceptual decline in healthy aging: The information degradation hypothesis. Neuroscience and Biobehavioral Reviews.

[CR27] Morales D, Pashler H (1999). No role for colour in symmetry perception. Nature.

[CR28] Norman JF, Higginbotham AJ (2020). Aging and the perception of global structure. PLOS ONE.

[CR29] Pilz KS, Äijälä JM, Manassi M (2020). Selective age-related changes in orientation perception. Journal of Vision.

[CR30] Posner MIC, Snyder RR, Davidson BJ (1980). Attention and detection of signals. Journal of Experimental Psychology: General.

[CR31] R Core Team (2016). *R: A language and environment for statisticall computing*.

[CR32] Rainville SJM, Kingdom FAA (2002). Scale invariance is driven by stimulus density. Vision Research.

[CR33] Roudaia E, Bennett PJ, Sekuler AB (2008). The effect of aging on contour integration. Vision Research.

[CR34] Roudaia E, Bennett PJ, Sekuler AB (2013). Contour integration and aging: The effects of element spacing, orientation alignment and stimulus duration. Frontiers in Psychology.

[CR35] Roudaia E, Sekuler AB, Bennett PJ (2014). Aging and the integration of orientation and position in shape perception. Journal of Vision.

[CR36] Sharman RJ, Gheorghiu E (2019). Orientation of pattern elements does not influence mirror-symmetry perception. Journal of Vision.

[CR37] Treisman AM, Gelade G (1980). A feature-integration theory of attention. Cognitive Psychology.

[CR38] Vallesi A, Tronelli V, Lomi F, Pezzetta R (2021). Age differences in sustained attention tasks: A meta-analysis. Psychonomic Bulletin & Review.

[CR39] van der Helm PA, Leeuwenberg ELJ (1996). Goodness of visual regularities: A nontransformational approach. Psychological Review.

[CR40] Wainwright JB, Scott-Samuel NE, Cuthill IC (2020). Overcoming the detectability costs of symmetrical coloration. Proceedings of the Royal Society B: Biological Sciences.

[CR41] Warnes, G. R., Bolker, B., & Lumley, T. (2015). gtools: Various R programming tools (R Package Version 3.5.0) [Computer software]. https://CRAN.R-project.org/package=gtools

[CR42] Wenderoth P (1996). The effects of dot pattern parameters and constraints on the relative salience of vertical bilateral symmetry. Vision Research.

[CR43] Westland S, Ripamonti C, Cheung V (2012). *Computational colour science using MATLAB*.

[CR44] Wickham H (2007). Reshaping data with the *reshape* package. Journal of Statistical Software.

[CR45] Wickham H (2009). *ggplot2: Elegant graphics for data analysis*.

[CR46] Wickham, H., Francois, R., Henry, L., & Mueller, K. (2017). *dplyr: A grammar of data manipulation* (R Package Version 0.7.2). https://CRAN.R-project.org/package=dplyr

[CR47] Wilson HR, Wilkinson F (2002). Symmetry perception: A novel approach for biological shapes. Vision Research.

[CR48] Wright D, Mitchell C, Dering BR, Gheorghiu E (2018). Luminance-polarity distribution across the symmetry axis affects the electrophysiological response to symmetry. NeuroImage.

[CR49] Wu CC, Chen CC (2014). The symmetry detection mechanisms are color selective. Scientific Reports.

[CR50] Wu CC, Chen CC (2017). The integration of color-selective mechanisms in symmetry detection. Scientific Reports.

[CR51] Wuerger, S. (2013). Colour constancy across the life span: evidence for compensatory mechanisms. *PLoS One, 8*. 10.1371/journal.pone.0063921 10.1371/journal.pone.0063921 PMC3648508 23667689

