# Supplementary material for: Mirror symmetry and aging: The role of stimulus figurality and attention to colour
Source: Atten Percept Psychophys. 2022 Sep 29;85(1):99–112. doi: 10.3758/s13414-022-02565-5 (PMC9816266; doi:10.3758/s13414-022-02565-5)
Supplement: Supplementary file 1 — (DOCX 24 kb) [file 13414_2022_2565_MOESM1_ESM.docx]

**Supplementary material: Statistical models**

As explained in the statistical analysis section, we used generalised linear mixed effect models (GLMMs) to analyse the data. Contrasts were set using the simple coding method – this means that every level of a factor is compared to a reference level, but the intercept corresponds to the grand mean. As explained in the methods section, we evaluated the contribution of the fixed factors and their interactions to the model by removing them and evaluating if such a reduced model became significantly poorer at explaining the variance in the dataset.

**Experiment 1: Detection of symmetry in wedge patterns**

Experiment 1 had the following within-subject factors: number of wedges (24 or 36 wedges), number of colours (2 or 3 colours, with 50% or 33% elements carrying the symmetry signal), attention to colour (either uncued or cued), and the type of colour symmetry - non-segregated, segregated or anti-symmetric. Simple coding was used for these factors, with reference level set as per list above (2 colours, uncued, non-segregated).

Due to an error in the generation of three-colour anti-symmetric images, this condition had to be removed prior to the analysis. To overcome this challenge, we fitted two generalised linear mixed effect models. The first one included number of wedges (24 or 36), number of colours (2 or 3), colour-symmetry type (only non-segregated and segregated, i.e. excluding anti-symmetric patterns) and attention (uncued or cued). The second included number of wedges (24 or 36), type of colour symmetry (including non-grouped anti-symmetric) and attention (uncued or cued) for two-colour patterns only.

**First GLMM analysis: How does the number of colours influence symmetry detection?**

The maximal model that could be adequately fitted to the dataset included random by-participant intercepts and interactive random slopes for attention and colour-symmetry type.

**Supplementary Table 1**. Estimated regression parameters, standard errors, z-values, p-values and standardised odds ratios for the best fitting GLMM in Experiment 1, on the dataset without anti-symmetric patterns.

| ***Fixed Effects*** | ***Estimate***  ***± SE*** | ***z value*** | ***p***  ***value*** | ***Odds ratio (95% CI)*** |
| --- | --- | --- | --- | --- |
| **Intercept** | 0.715 ± 0.043 | 16.810 | <.001*** | 2.04  (1.88-2.22) |
| **Main effects** |  |  |  |  |
| Colour symmetry | -0.815 ± 0.074 | -11.030 | <.001*** | 0.44  (0.38-0.51) |
| Number of wedges | -0.061 ± 0.041 | -1.483 | 0.138 | 0.94  (0.87-1.02) |
| Number of colours | 0.043 ± 0.041 | 1.050 | 0.294 | 1.04  (0.96-1.13) |
| Attention | 0.232 ± 0.085 | 2.738 | 0.006** | 1.26  (1.07-1.49) |
| **Interactions** |  |  |  |  |
| Number of colours by Colour-symmetry | 0.238 ± 0.082 | 2.902 | 0.004*** | 1.27  (1.08-1.49) |
| Number of colours by number of wedges | 0.224 ± 0.082 | 2.743 | 0.006** | 1.25  (1.07-1.47) |
| Colour-symmetry by Attention | -1.121 ± 0.171 | -6.566 | <.001*** | 0.33  (0.23-0.46) |
| Number of colours by Attention | -0.164 ± 0.082 | -2.005 | 0.045* | 0.85  (0.72-1.00) |
| Colour-symmetry by Number of wedges | 0.234 ± 0.082 | 2.866 | 0.004** | 1.26  (1.08-1.48) |
| Number of colours by Number of wedges by Colour Symmetry | -0.381 ± 0.163 | -2.335 | 0.0195* | 0.68  (0.50-0.94) |

The four-way interaction between number of wedges, number of colours, colour-symmetry and perceptual condition (with vs without attention) did not contribute significantly to this fit (χ^2^(1)=0.100, p=.752). This was also the case for three-way interactions between number of colours, wedges and attention (χ^2^(1)=2.051, p=.152), number of wedges, colour-symmetry and attention (χ^2^(1)=1.235, p=.266), nor number of colours, colour-symmetry and attention (χ^2^(1)=0.033, p=0.856).

**Second GLMM analysis: How are anti-symmetric wedge patterns processed?**

In the second model, we performed GLMMs on 2-colour wedges only. This allowed us to include anti-symmetric stimuli, which were only available for 2-colour patterns. The maximal model that could be fitted while maintaining a satisfactory goodness of fit included only one random effect (by-participant intercepts) and the fixed effects of colour-symmetry, number of wedges and attention and their interactions.

**Supplementary Table 2**. Estimated regression parameters, standard errors, z-values, p-values and standardised odds ratios for the best fitting GLMM in Experiment 1, on the dataset with anti-symmetric patterns, but without 3-colour patterns.

| ***Fixed Effects*** | ***Estimate ± SE*** | ***z value*** | ***p***  ***value*** | ***Odds ratio (95% CI)*** |
| --- | --- | --- | --- | --- |
| **Intercept** | 0.541 ± 0.036 | 15.196 | <.001*** | 1.72  (1.60-1.84) |
| **Simple contrasts** |  |  |  |  |
| Segregated vs. non-segregated | 0.896 ± 0.058 | 15.343 | <.001*** | 2.45  (2.18-2.75) |
| Anti-symmetric vs. non-segregated | 0.052 ± 0.053 | 0.974 | .330 | 1.05  (0.95-1.17) |
| Number of wedges | -0.154 ± 0.046 | -3.347 | <.001*** | 0.86  (0.78-0.94) |
| Attention | 0.174 ± 0.061 | 2.834 | .005** | 1.19  (1.06-1.34) |
| **Interactions** |  |  |  |  |
| Segregation of pattern by number of wedges | -0.419 ± 0.115 | -3.626 | <.001*** | 0.66  (0.52-0.83) |
| Anti-symmetry of pattern by number of wedges | -0.162 ± 0.107 | -1.520 | 0.129 | 0.85  (0.69-1.05) |
| Segregation of pattern by Attention | 1.030 ± 0.116 | 8.840 | <.001*** | 2.80  (2.23-3.52) |
| Anti-symmetry of pattern by Attention | 0.206 ± 0.107 | 1.936 | .053 | 1.23  (1.00-1.52) |

**Experiment 2: Symmetry detection for dot and wedge patterns in younger and older adults**

The following within-subject factors were entered as fixed effects: stimulus type (wedge vs. dot patterns), colour-symmetry type (non-segregated; segregated; randomly segregated; colour-grouped anti-symmetric; non-grouped anti-symmetric) and attention (uncued vs. cued). Age group (younger vs. older) was the between-subject fixed effect. Random effects of by-participant intercepts and by-participant slopes for stimulus type and attention were also entered into the model. This was the maximal model that could be reliably fitted, and both attention (χ^2^(2)=23.055, p<.001) and stimulus type (χ^2^(3)=40.722, p<.001) random slopes made a significant difference when compared with only by-participant random intercepts. For colour-symmetry type, we evaluated four contrasts: non-segregated vs., in turn, segregated, random segregated, colour-grouped anti-symmetric and non-grouped anti-symmetric patterns.

**Table 3.** Estimated regression parameters, standard errors, z-values, p-values and standardised odds ratios for the best fitting GLMM in Experiment 2.

| Fixed Effects | Estimate  ± SE | z value | p  value | Odds ratio  (95% CI) |
| --- | --- | --- | --- | --- |
| **Intercept** | 0.907 ± 0.053 | 17.221 | <.001*** | 2.477  (2.234-2.747) |
| **Main effects** |  |  |  |  |
| Segregated vs. non-segregated | 0.477 ± 0.047 | 10.236 | <.001*** | 1.612  (1.471-1.766) |
| Random segregated vs. non-segregated | 0.278 ± 0.045 | 6.144 | <.001*** | 1.320  (1.208-1.443) |
| Grouped anti-symmetric vs. non-segregated | -0.040 ± 0.044 | -0.911 | 0.363 | 0.961  (0.882-1.047) |
| Non-grouped anti-symmetric vs. non-segregated | -0.381 ± 0.043 | -8.847 | <.001*** | 0.683  (0.628-0.743) |
| Stimulus Type | -0.440 ± 0.055 | -7.933 | <.001*** | 0.644  (0.578-0.718) |
| Age Group | -0.272 ± 0.105 | -2.590 | .010 ** | 0.762  (0.620-0.936) |
| Attention | 0.087 ± 0.049 | 1.778 | 0.0754 | 1.091  (0.991-1.201) |
| **Interactions** |  |  |  |  |
| Segregation of pattern by Stimulus type | 0.886 ± 0.093 | 9.515 | <.001*** | 2.426  (2.021-2.912) |
| Random segregation by Stimulus type | 0.675 ± 0.090 | 7.464 | <.001*** | 1.964  (1.645-2.345) |
| Grouped anti-symmetry of pattern by Stimulus type | 0.578 ± 0.087 | 6.626 | <.001*** | 1.783  (1.503-2.116) |
| Non-grouped anti-symmetry of pattern by Stimulus type | 0.210 ± 0.086 | 2.442 | 0.015* | 1.234  (1.042-1.460) |
| Segregation of pattern by Attention | 0.417 ± 0.093 | 4.471 | <.001*** | 1.517  (1.264-1.821) |
| Random segregation of pattern by Attention | 0.149 ± 0.090 | 1.653 | 0.098 | 1.161  (0.973-1.386) |
| Grouped anti-symmetry of pattern by Attention | -0.073 ± 0.087 | -0.837 | 0.402 | 0.929  (0.783-1.103) |
| Non-grouped anti-symmetry of pattern by Attention | -0.528 ± 0.086 | -6.137 | <.001*** | 0.590  (0.498-0.698) |
| Stimulus type by Age group | -0.036 ± 0.110 | -0.326 | 0.744 | 0.965  (0.777-1.198) |
| Age group by attention | -0.005 ± 0.098 | -0.046 | 0.963 | 0.995  (0.821-1.206) |
| Stimulus type by attention | 0.072 ± 0.057 | 1.263 | 0.207 | 1.075  (0.961-1.201) |
| Stimulus type by Attention by Age Group | 0.511 ± 0.112 | 4.543 | <.001*** | 1.668  (1.338-2.079) |
| Segregation by Stimulus Type by Attention | 0.397 ± 0.186 | 2.138 | 0.033* | 1.488  (1.034-2.141) |
| Random segregation by Stimulus Type by Attention | 0.497 ± 0.180 | 2.756 | 0.006** | 1.643  (1.154-2.339) |
| Grouped anti-symmetry by Stimulus Type by Attention | -0.002 ± 0.174 | -0.011 | 0.991 | 0.998  (0.710-1.404) |
| Non-grouped anti-symmetry by Stimulus Type by Attention | -0.618 ± 0.171 | -0.360 | 0.718 | 0.940  (0.672-1.315) |

The four-way interaction did not contribute significantly to the model (χ^2^(4)=5.5398, p=0.2363), and neither did the following three-way interactions: colour-symmetry type by attention by age group (χ^2^(4)=4.8038, p=0.308) and colour-symmetry by stimulus type by age group (χ^2^(4)=5.596, p=0.2314). Thus, our final model involved a three-way interaction between stimulus type, attention and age group, as well as the three-way interaction involving colour symmetry type, stimulus type and attention. As colour-symmetry type and age group were not involved in the same three-way interaction, we evaluated if we could leave out their two-way interaction. We discovered that it did not contribute significantly to the model (χ^2^(4)=4.9597, p=0.2915).
